# Correlation between Peripheral T Cell Subsets and the Activity of Thyroid-Associated Ophthalmopathy

**DOI:** 10.1155/2022/2705650

**Published:** 2022-03-08

**Authors:** Hong Hu, Liang Liang, Qian Ge, Xing Jiang, Zhizheng Fu, Chun Liu, Jian Long

**Affiliations:** ^1^Department of Endocrinology, The First Affiliated Hospital of Chongqing Medical University, Chongqing 400016, China; ^2^Chongqing Key Laboratory of Ophthalmology, and Chongqing Eye Institute, The First Affiliated Hospital of Chongqing Medical University, Chongqing 400016, China

## Abstract

**Introduction:**

Thyroid-associated ophthalmopathy (TAO) is the most common orbital immunological disease in adults. T cells play an important role in the pathogenesis of TAO. However, our knowledge of the circulating T cell subsets in TAO is limited.

**Objective:**

To investigate the circulating T cell subsets in TAO and the correlations between them and the activity of TAO.

**Methods:**

Thirty-eight TAO patients (19 active and 19 nonactive) were enrolled. The absolute number and percentage of total lymphocytes, CD3+T cells, CD4+T cells, CD8+ T cells, CD3+CD4-CD8-T cells (DNT cells), and CD3+CD4+CD8+ T cells (DPT cells) in peripheral blood were measured by flow cytometer.

**Results:**

TAO patients were divided into the active group and the nonactive group by the clinical activity score (CAS). The mean CAS was 4 ± 1.11 in the active group and 1.47 ± 0.61 in the nonactive group. No statistical differences were found in gender, age, and the levels of FT3, FT4, TSH, and TRAb between the two groups. The percentage of DNT cells was lower in the active group than in the non-active group, and it was negatively correlated with CAS (*r* = −0.349, *P*=0.032), but not the absolute number. The CD4/CD8 ratio, the absolute number and percentage of CD3+ T cells, CD4+ T cells, CD8+ T cells, and DPT cells did not differ between the two groups.

**Conclusion:**

In the present study, we found the percentage of DNT cells was significantly lesser in the active TAO than in the nonactive TAO, and it was negatively correlated with the activity of the TAO. It suggests that DNT cells may involve in the immunopathogenesis of TAO and can serve as a clinical biomarker of the disease activity.

## 1. Introduction

Thyroid-associated ophthalmopathy (TAO) is the most common orbital disease in adults [1]. Patients with serious TAO are at risk of blindness and disability, which occurs in 5–8% of all cases and is so-called sight-threatening TAO [2]. The basic process of TAO includes the infiltration of immune cells in orbital tissues, the proliferation and differentiation of fibroblasts (OFs), and adipogenesis [3, 4]. Of which, the lymphocyte-mediated immunity is thought to be the initiating factor of TAO [5]. Among these lymphocytes, T cells are thought to be the main effector cells, which produce a variety of adhesion molecules and cytokines, mediate the recruitment of more lymphocytes into orbital tissues, and promote the proliferation and differentiation of orbital fibroblasts (OFs) [6, 7].

Several T cell subsets have been found in the orbit of TAO. Of these, CD4 +T cells and CD8 +T cells are the most prevalent T cell subsets [8, 9]. Previous studies have shown that the total number of infiltrating cells in orbit correlates with the clinical activity score (CAS) of TAO [10]. However, in clinical, orbital tissue specimens are very difficult to obtain, but peripheral blood samples are easier to get. Little is known about the peripheral blood T cell subsets, much less the relationship between T cell subsets and the activity of TAO.

Recently CD3+CD4-CD8- T cells, so-called double-negative T cells (DNT cells), and CD3+CD4+CD8+T cells, known as double-positive T cells (DPT cells), were also found in the orbit of TAO [8, 11]. While the functions of DNT and DPT cells in TAO remain unclear yet.

Thus, in order to gain a deeper understanding of the immune mechanism responsible for TAO, we measured T cell subsets in the peripheral blood of TAO patients, especially DNT and DPT cells, and analyzed the relationship between them and the activity of TAO.

## 2. Materials and Methods

### 2.1. Subjects

Between January 2020 and January 2021, TAO patients who visited the First Affiliated Hospital of Chongqing Medical University were enrolled in our study. The inclusion criteria were as follows: (a) TAO patients; and (b) age between >18 years and <70 years. The exclusion criteria were as follows: (a) patients who have a history of other autoimmune diseases, such as systemic lupus erythematosus; (b) treatment with glucocorticoids or other immunosuppressants within six months before the study; and (c) patients with significant active infection, tuberculosis, or those complicated by macrophage activation syndrome.

All subjects recruited signed the consent form and agreed to take part in the present study. The study was approved by the First Affiliated Hospital of Chongqing Medical University Ethical Committee.

### 2.2. The Diagnosis and Assessment of TAO

The diagnosis of TAO is based on the consensus statement of the European Group on Graves' Orbitopathy criteria [12]. The activity of TAO was evaluated by the CAS system, which includes seven reference items chemosis, redness of conjunctiva and eyelid, swelling of the eyelid, swelling of the caruncle, spontaneous ache behind the eyeball, and ache on attempted upgaze. One symptom added one point, and the CAS score ≥3 was classified as an active stage. The mean CAS was the average CAS of two eyes in one patient.

### 2.3. Laboratory Tests

FT3, FT4, and TSH were determined with the electrochemiluminescence method (Unicel DxI 800 Immunoassay System, Beckman Coulter, USA). TR-Abs were examined using chemiluminescence immunoassay (Cobas601, Roche, Germany).

### 2.4. Flow Cytometry

The absolute number of total lymphocytes, CD3+ T cells, CD4+ T cells, CD8+ T cells, DNT cells, and DPT cells were measured by flow cytometry. The following FITC monoclonal antibodies were used: anti-CD3, anti-CD4-PC7, and anti-CD8-APC-Cy7 (QuantoBio, China). 50 µL of peripheral blood cells were incubated with 20 µl antibodies for 15 minutes in the dark at room temperature. After incubation, red blood cells were lysed with a lysing solution for 15 min. Then, cell suspensions were acquired in a flow cytometer (Beckman Coulter, Navios, USA).

### 2.5. Statistical Analysis

SPSS 22.0 package software was used to analyse all data. Continuous data are given as the mean ± standard deviation. Categorical variables are reported as numbers. Means of continuous variables were compared using the unpaired *t*-test or Mann–Whitney test (when the data were not normally distributed). Spearman's rank correlation test was used to evaluate the correlations between T cells and CAS. A *P*value <0·05 was considered to be significant.

## 3. Results

### 3.1. Patient Demographic Data and Clinical Activity Score

Thirty-eight TAO patients (22 females and 16 males) with a mean age of 47.74 ± 8.94 years (range 26–66 years) were enrolled in this study, including 19 active and 19 nonactive patients. As shown in Table 1, the mean CAS was higher in the active group than in the nonactive group with a statistical difference. While no significant differences were found in terms of gender, age, the levels of FT3, FT4, TSH, and TRAb (all *P* > 0.05) between the two groups.

### 3.2. T Cell Subsets in the Active and Nonactive Group

As shown in Table 2, there were no significant differences in the absolute number of total lymphocytes, CD3+ T cells, CD4+ T cells, CD8+ T cells, DNT cells, as well as DPT cells between the active group and the nonactive group (all *P* > 0.05). The CD4/CD8 ratio did not differ between the two groups too (*P* > 0.05). And there were no significant differences in the percentage of total lymphocytes, CD3+ T cells, CD4+ T cells, CD8+ T cells, and DPT cells between the two groups (all *P* > 0.05).While the percentage of DNT cells was lower in the active group compared to the nonactive group (*P*=0.024).

### 3.3. Correlations between DNT Cells and CAS

The association between DNT cells and CAS was further analyzed. As shown in Table 3, the percentage of DNT cells was negatively correlated with CAS (*r* = −0.349, *P*=0.032).

## 4. Discussion

The stability and balance of T cell subsets is an important factor for the body's immune function [13]. In the present study, we assessed various T cell subsets, including total lymphocytes, CD3+ T cells, CD4+T cells, CD8+T cells, DNT cells, and DPT cells in patients with TAO. We found that only the percentage of DNT cells was significantly lower in the active TAO patients than in the nonactive TAO patients, and it was negatively correlated with the activity of TAO. Our results suggest that DNT cells may involve in the pathogenesis of TAO and can be a clinical biomarker of disease activity. This is the first study to demonstrate the potential role of DNT cells in TAO.

DNT cells account for 1–3% of total lymphocytes in peripheral blood and play an important role in various diseases' immunopathogenesis [14–16]. In patients with antineutrophil cytoplasmic autoantibody (ANCA)-associated vasculitis (AAV), the percentage of DNT cells was elevated along with renal damage [17]. Similarly, in systemic lupus erythematosus (SLE), DNT cells were also found to be elevated and positively correlated with the disease activity [18, 19]. In contrast to that, in our study, the percentage of DNT cells decreased in the active TAO patients and had a negative correlation with the activity of TAO. The most possible explanation for this opposite phenomenon may be the different pathophysiological processes. In SLE, autoimmune inflammation mainly leads to tissue and organ damage [20, 21], but in TAO it also leads to the proliferation and differentiation of fibroblasts and preadipocytes [3, 22]. The other reason may be the different roles of DNT cells in different diseases. DNT cells not only induce inflammation by producing various cytokines, such as interleukin (IL)-2, IL-4, TNFa, and IL-17A [23, 24], but also regulate inflammation by suppressing syngeneic CD4+ or CD8+ T cells [25]. Interestingly, the correlation was not found in the absolute number of DNT cells and CAS in our study, but a decreased trend of the absolute number of DNT cells in active TAO was observed. The limited samples may be the real reason.

CD4+ and CD8+ T cells are the main effector cells in TAO. In our study, both the absolute number and the percentage of CD4+ T cells, CD8+ T cells, and the CD4/CD8 ratio did not differ between the active and the nonactive group. In peripheral blood, CD4+T cells and CD8+T cells are the main lymphocytes with a huge number [26].Thus, a small amount of cells migrating and infiltrating in the orbit may not be enough to change the absolute number and the percentage of these cells. As the same, the absolute number and percentage of total lymphocytes did not differ between the active and the non-active group, which is consistent with the report of Ionni [27].

DPT cells are mainly present in the thymus and further differentiate into CD4+ or CD8+ T cells [28]. Recently, DPT cells were also found in the orbital connective tissue of patients with active TAO by immunohistochemical analysis [26]. While in our study, no significant difference was found in peripheral blood DPT cells between the active and the nonactive group. Thus, the real function of DPT cells in TAO needs further investigation.

There were still some limitations. Firstly, it is a small sample study, so our results need to be replicated in research with a large and more patient population. Secondly, our research is a clinical study which does not involve the function of these T cells, and much fewer mechanisms. Future animal or human experiments are required to clarify the potential function and mechanism in the future.

## 5. Conclusion

In the present study, we found that the percentage of DNT cells were significantly lesser in the active TAO patients than in the nonactive patients and was negatively correlated with the activity of TAO. These findings suggest that DNT cells may be involved in the pathogenesis of TAO and can serve as a clinical biomarker of disease activity.

## Figures and Tables

**Table 1:** Demographic and clinical characteristics of patients with TAO.

	Active TAO (*n* = 19)	Non-active TAO (*n* = 19)	*P*
Mean CAS	4 ± 1.11	1.47 ± 0.61	0#
Male/Female(*n*/*n*)	11/8	5/14	0.052#
Age,years	49.84 ± 8.5 years	45.63 ± 9.09	0.149^*∗*^
FT3 (pg/ml)	3.55 ± 0.68	3.26 ± 0.37	0.182^*∗*^
FT4 (ng/dl)	0.90 ± 0.35	0.78 ± 0.14	0.209#
TSH (mU/L)	1.45 ± 1.95	3.1 ± 2.74	0.077#
TRAb(IU/l)	11.14 ± 10.34	11.35 ± 11.19	1.0#

Data represented as mean ± SD. ^*∗*^:The Unpaired *t*-test; #:Mann–Whitney Test; *P*-value <0.05 was considered to be significant. CAS: clinical activity score.

**Table 2:** T cell subsets in TAO with different activity.

	Active TAO (*n* = 22)	Non-active TAO (*n* = 19)	*P*
Total lymphocytes(n/ul)	2316.32 ± 488.62	2275.68 ± 770.50	0.85^*∗*^
CD3^+^ T cell(n/ul)	1680.95 ± 351.53	1688 ± 594.73	0.97^*∗*^
CD4^+^ T cell(n/ul)	1024.11 ± 231.02	960.89 ± 390.18	0.24#
CD8^+^ T cell(n/ul)	592.63 ± 201.14	606.11 ± 263.72	0.86^*∗*^
DPT cell(n/ul)	10.79 ± 15.34	8.05 ± 5.38	0.78#
DNT cell(n/ul)	82.47 ± 49.55	126.84 ± 95.75	0.17#
CD4/CD8 ratio	1.9263 ± 0.74	1.7426 ± 0.72	0.44^*∗*^
Total lymphocytes(%)	32.09% ± 8.32%	36.33% ± 8.48%	0.25#
CD3^+^ T cell(%)	72.83% ± 5.82%	73.99% ± 6.19%	0.56^*∗*^
CD4^+^ T cell(%)	44.42% ± 4.78%	42.17% ± 7.27%	0.27^*∗*^
CD8^+^ T cell(%)	25.69% ± 7.66%	26.7% ± 7.82%	0.69^*∗*^
DPT cell(%)	0.45% ± 0.6%	0.37% ± 0.23%	0.66#
DNT cell(%)	3.44% ± 1.64%	5.43% ± 3.22%	0.02#

^*∗*^:The Unpaired *t*-test; #:Mann–Whitney Test; *P*-value <0.05 was considered to be significant.

**Table 3:** Correlation between the CAS activity and DNT cells.

	Mean CAS	
	*r* value	*P*
DNT cell(%)	−0.349	0.032

CAS: clinical activity score. Spearman's rank correlation test was used to evaluate the correlations between DNT cells and CAS. *P*-value <0.05 was considered to be significant.

## Data Availability

The data supporting this research article are available from the corresponding author on reasonable request.

## References

[1] Taylor P. N., Zhang L., Lee R. W. J. (2020). New insights into the pathogenesis and nonsurgical management of Graves orbitopathy. *Nature Reviews Endocrinology*.

[2] Tanda M. L., Piantanida E., Liparulo L. (2013). Prevalence and natural history of Graves’ orbitopathy in a large series of patients with newly diagnosed graves’ hyperthyroidism seen at a single center. *Journal of Clinical Endocrinology & Metabolism*.

[3] Khong J. J., McNab A. A., Ebeling P. R., Craig J. E., Selva D. (2016). Pathogenesis of thyroid eye disease: review and update on molecular mechanisms. *British Journal of Ophthalmology*.

[4] Wang Y., Smith T. J. (2014). Current concepts in the molecular pathogenesis of thyroid-associated ophthalmopathy. *Investigative Opthalmology & Visual Science*.

[5] Huang Y., Fang S., Li D., Zhou H., Li B., Fan X. (2019). The involvement of T cell pathogenesis in thyroid-associated ophthalmopathy. *Eye*.

[6] Pappa A., Lawson JM., Calder V., Fells P., Lightman S. (2000). T cells and fibroblasts in affected extraocular muscles in early and late thyroid associated ophthalmopathy. *British Journal of Ophthalmology*.

[7] Fang S., Huang Y., Wang N. (2019). Insights into local orbital immunity: evidence for the involvement of the Th17 cell pathway in thyroid-associated ophthalmopathy. *Journal of Clinical Endocrinology & Metabolism*.

[8] Hai Y. P., Lee A. C. H., Frommer L., Diana T., Kahaly G. J. (2020). Immunohistochemical analysis of human orbital tissue in Graves’ orbitopathy. *Journal of Endocrinological Investigation*.

[9] Grubeck-Loebenstein B., Trieb K., Sztankay A., Holter W., Anderl H., Wick G. (1994). Retrobulbar T cells from patients with Graves’ ophthalmopathy are CD8+ and specifically recognize autologous fibroblasts. *Journal of Clinical Investigation*.

[10] Rotondo Dottore G., Torregrossa L., Caturegli P. (2018). Association of T and B cells infiltrating orbital tissues with clinical features of graves orbitopathy. *JAMA Ophthalmology*.

[11] He K. (2005). Immunohistochemical analysis of orbital connective tissue specimens of patients with active Graves ophthalmopathy. *Current Eye Research*.

[12] Bartalena L., Baldeschi L., Boboridis K. (2016). The 2016 European thyroid association/European group on graves’ orbitopathy guidelines for the management of graves’ orbitopathy. *European Thyroid Journal*.

[13] Taylor J. M., Fahey J. L., Detels R., Giorgi J. V. (1989). CD4 percentage, CD4 number, and CD4:CD8 ratio in HIV infection: which to choose and how to use. *Journal of Acquired Immune Deficiency Syndromes*.

[14] Li Y., Dong K., Fan X. (2020). DNT cell-based immunotherapy: progress and applications. *Journal of Cancer*.

[15] Kokuina E. (2020). Determination of reference values for double-negative T lymphocytes in Cuban adults. *MEDICC review*.

[16] Brandt D., Hedrich C. M. (2018). TCR*αβ*+ CD3+ CD4− CD8− (double negative) T cells in autoimmunity. *Autoimmunity Reviews*.

[17] Qin Y., Wang Y., Wu Y. (2021). Double-negative T cells are absolutely elevated in patients with antineutrophil cytoplasmic autoantibody-associated vasculitis. *Molecular Immunology*.

[18] El-Sayed Z. A., El-Owaidy R. H., Mohamed N. L., Shehata B. A. (2018). Alpha beta double negative T cells in children with systemic lupus erythematosus: the relation to disease activity and characteristics. *Modern Rheumatology*.

[19] Shaltout A. S., Sayed D., Badary M. S. (2016). Effect of IL6 and IL23 on double negative T cells and anti ds-DNA in systemic lupus erythematosus patients. *Human Immunology*.

[20] Ghodke-Puranik Y., Niewold T. B. (2015). Immunogenetics of systemic lupus erythematosus: a comprehensive review. *Journal of Autoimmunity*.

[21] Mak A., Isenberg D. A., Lau C.-S. (2013). Global trends, potential mechanisms and early detection of organ damage in SLE. *Nature Reviews Rheumatology*.

[22] Smith T. J. (2015). TSH-receptor-expressing fibrocytes and thyroid-associated ophthalmopathy. *Nature Reviews Endocrinology*.

[23] Crispín J. C., Oukka M., Bayliss G. (2008). Expanded double negative T cells in patients with systemic lupus erythematosus produce IL-17 and infiltrate the kidneys. *The Journal of Immunology*.

[24] Lu Y., Wang X. J., Yan W. M. (2012). Q.Ning. Liver TCR*γδ*(+) CD3(+) CD4(-) CD8(-) T cells contribute to murine hepatitis virus strain 3-induced hepatic injury through a TNF-*α*-dependent pathway. *Molecular Immunology*.

[25] Chen W., Ford M. S., Young K. J., Zhang L. (2004). The role and mechanisms of double negative regulatory T cells in the suppression of immune responses. *Cellular and Molecular Immunology*.

[26] Xu D., Wu Y. Y., Gao C. (2020). Characteristics of and reference ranges for peripheral blood lymphocytes and CD4 + T cell subsets in healthy adults in Shanxi Province, North China. *Journal of International Medical Research*.

[27] Ionni I., Rotondo Dottore G., Marinò M. (2018). Peripheral T and B lymphocytes do not correlate with Graves’ orbitopathy. *Journal of Endocrinological Investigation*.

[28] Overgaard N. H., Jung J.-W., Steptoe R. J., Wells J. W. (2015). CD4+/CD8+double-positive T cells: more than just a developmental stage?. *Journal of Leukocyte Biology*.

